# Uremic toxins mediate kidney diseases: the role of aryl hydrocarbon receptor

**DOI:** 10.1186/s11658-024-00550-4

**Published:** 2024-03-15

**Authors:** Hongyan Xie, Ninghao Yang, Chen Yu, Limin Lu

**Affiliations:** 1 Department of Nephrology, Tongji Hospital, Tongji University School of Medicine, 389 Xincun Road, Shanghai, 200065 China; 2 Department of Physiology and Pathophysiology, School of Basic Medical Sciences, Fudan University, 138 Yixueyuan Road, Shanghai, 200032 China

**Keywords:** Uremic toxins, Aryl hydrocarbon receptor, Acute kidney injury, Chronic kidney disease, Diabetic nephropathy

## Abstract

Aryl hydrocarbon receptor (AhR) was originally identified as an environmental sensor that responds to pollutants. Subsequent research has revealed that AhR recognizes multiple exogenous and endogenous molecules, including uremic toxins retained in the body due to the decline in renal function. Therefore, AhR is also considered to be a uremic toxin receptor. As a ligand-activated transcriptional factor, the activation of AhR is involved in cell differentiation and senescence, lipid metabolism and fibrogenesis. The accumulation of uremic toxins in the body is hazardous to all tissues and organs. The identification of the endogenous uremic toxin receptor opens the door to investigating the precise role and molecular mechanism of tissue and organ damage induced by uremic toxins. This review focuses on summarizing recent findings on the role of AhR activation induced by uremic toxins in chronic kidney disease, diabetic nephropathy and acute kidney injury. Furthermore, potential clinical approaches to mitigate the effects of uremic toxins are explored herein, such as enhancing uremic toxin clearance through dialysis, reducing uremic toxin production through dietary interventions or microbial manipulation, and manipulating metabolic pathways induced by uremic toxins through controlling AhR signaling. This information may also shed light on the mechanism of uremic toxin-induced injury to other organs, and provide insights into clinical approaches to manipulate the accumulated uremic toxins.

**Graphical Abstract:**

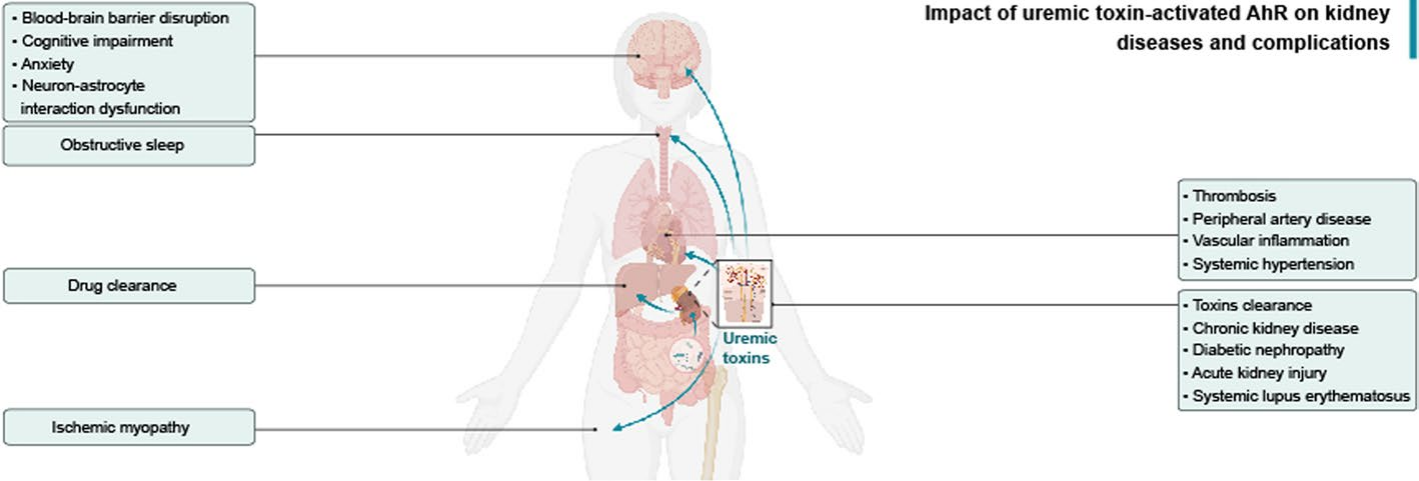

## Introduction

The kidney is the organ that excretes metabolic waste products, including creatinine, urea and uric acid, from the body. When metabolic waste products cannot be appropriately eliminated by the kidney, the accumulation of uremic toxins and disruption of the body’s internal environmental homeostasis are hazardous to all tissues and organs [1]. In recent years, the identification of endogenous uremic toxin receptors has opened the door to research on the precise role and molecular mechanism of uremic toxins in the tissue and organ, leading to the emergence of valuable insights [2].

Aryl hydrocarbon receptor (AhR) is an important receptor of uremic toxins. AhR was initially identified as an environmental sensor that responds to pollutants, including halogenated aromatic hydrocarbons and polycyclic aromatic hydrocarbons [3]. Growing evidence has suggested that AhR not only is a receptor for xenobiotics but can also be activated by various physiological ligands, such as metabolites derived from the host, gut microbiota or natural plants. Numerous studies have demonstrated that AhR activation is widely involved in cell differentiation, cellular senescence, lipid metabolism, intestinal balance, immune response and fibrogenesis [4–7]. Recent studies have indicated that AhR activation by the accumulation of uremic toxins may be implicated in various kidney diseases, including chronic kidney disease (CKD), CKD-associated complications, diabetic nephropathy (DN), acute kidney injury (AKI) and systemic lupus erythematosus (SLE) [8, 9]. Reducing uremic toxins by improving their clearance or inhibiting their production benefits clinical treatment outcomes [9]. However, these therapies possess inherent advantages and limitations that may contribute to poor outcomes for patients with kidney diseases. Targeting AhR with agonists or antagonists has shown promising initial efficacy in various kidney disease models [2]. Given the importance of understanding the effects of AhR activation by uremic toxins on kidney diseases and complications, this review summarizes the recent understanding of the mechanisms of uremic toxin-activated AhR signaling pathways and their effects on different renal diseases and also simply discusses current therapeutic strategies for targeting both uremic toxins and AhR activation.

## Uremic toxins

During the development of CKD, some metabolic waste products (including uremic toxins) are retained in the circulation and tissues due to a decreased glomerular filtration rate (GFR) and renal structural and physiological dysfunction [1]. Many uremic toxins are products of dietary constituents. For instance, p-cresyl sulfate (PCS) is derived from tyrosine; kynurenine (KYN) and indoxyl sulfate (IS) are derived from tryptophan (Trp); and trimethylamine-N-oxide (TMAO) is derived from dietary fish, red meat and eggs [10].

**Fig. 1:**
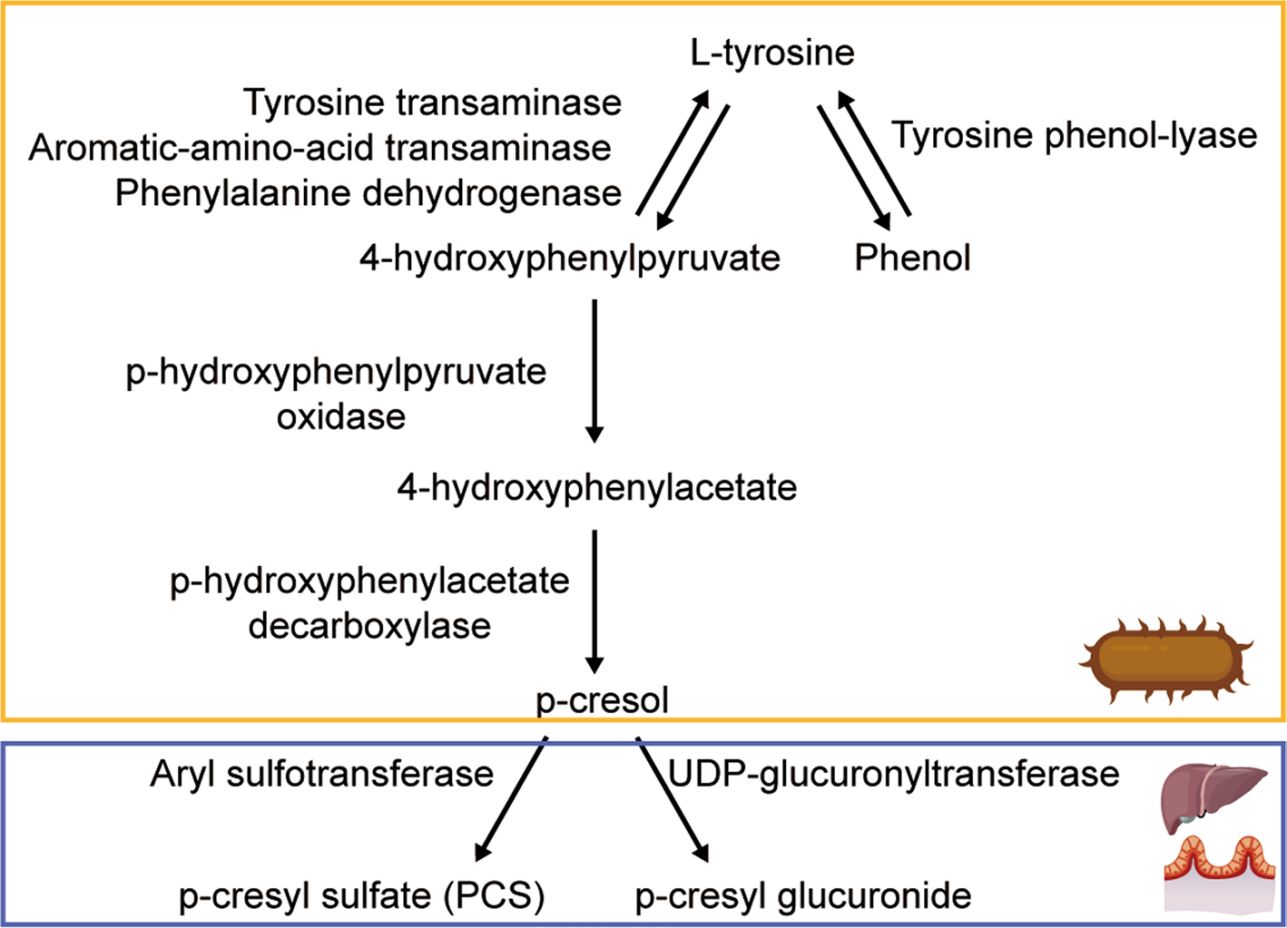
Metabolic pathway of tyrosine into p-cresyl sulfate (PCS). l-tyrosine can be reversibly converted to 4-hydroxyphenylpyruvate by tyrosine transaminase, aromatic-amino-acid transaminase or phenylalanine dehydrogenase. 4-Hydroxyphenylpyruvate is the precursor of 4-hydroxyphenylacetate, which is catalyzed by p-hydroxyphenylpyruvate oxidase, and can subsequently be decarboxylated to p-cresol by p-hydroxyphenylacetate decarboxylase. The majority of p-cresol is sulfated into the PCS by aryl sulfotransferases, and a small fraction is metabolized to p-cresyl glucuronide by UDP-glucuronyltransferases. The processes marked by the yellow box occur in the gut microbiota, and the processes marked by the blue box occur in the gut mucosa and liver of the host

L-tyrosine can be reversibly converted to phenol by tyrosine phenol-lyase [11]. In addition, L-tyrosine can also be reversibly converted to 4-hydroxyphenylpyruvate by tyrosine transaminase [12], aromatic-amino-acid transaminase [13] or phenylalanine dehydrogenase [14]. 4-Hydroxyphenylpyruvate is the precursor of 4-hydroxyphenylacetate, which is catalyzed by p-hydroxyphenylpyruvate oxidase [12], and can subsequently be decarboxylated to p-cresol by p-hydroxyphenylacetate decarboxylase [15]. These enzymes are present in the gut microbiota. The majority of p-cresol is sulfated into the PCS by aryl sulfotransferases [16], and a small fraction is metabolized to p-cresyl glucuronide by UDP-glucuronyltransferases in the gut mucosa and liver [17, 18] (Fig. 1).

**Fig. 2:**
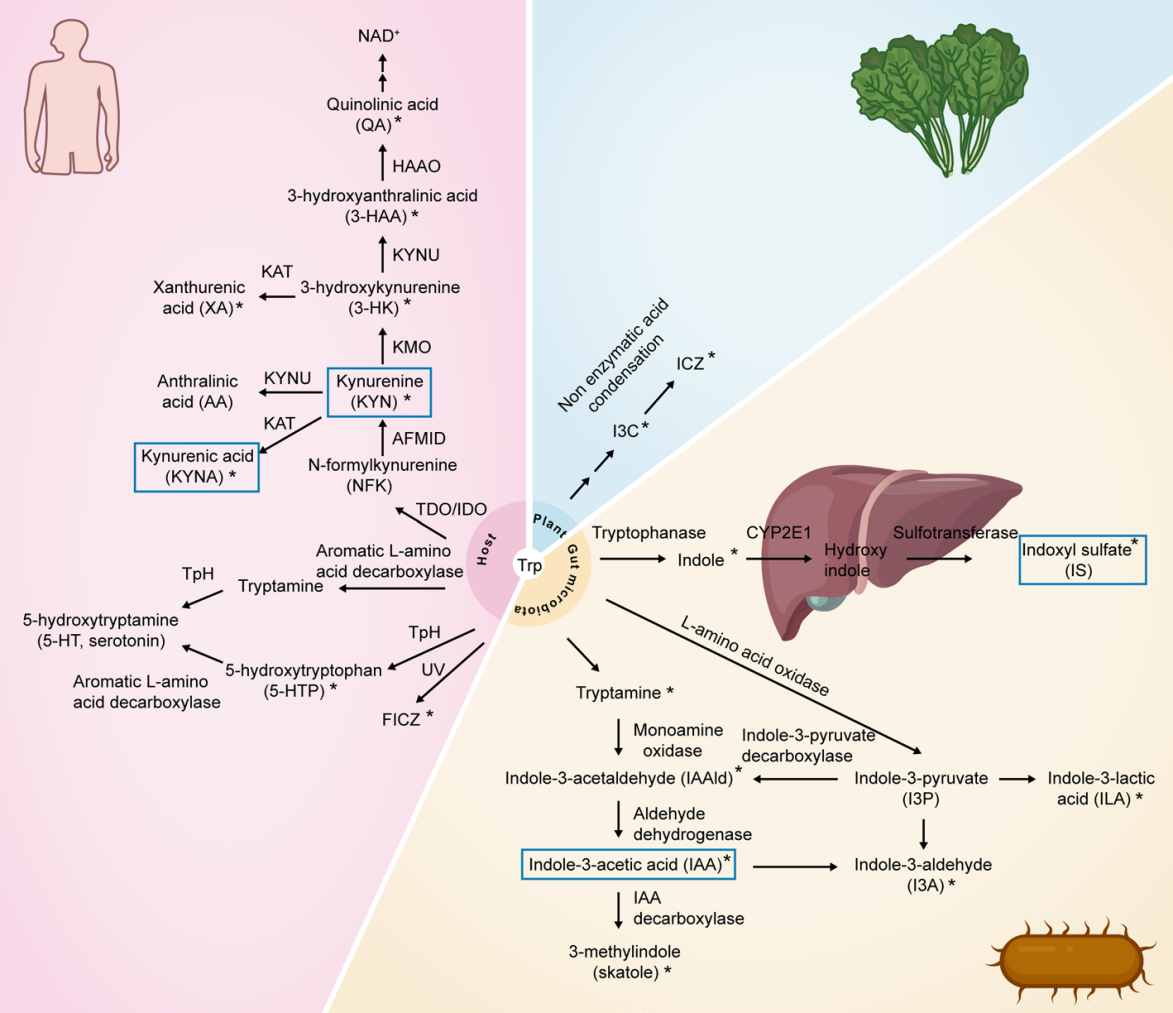
Metabolic pathway of tryptophan (Trp). Trp is mainly degraded through three known metabolic pathways that can be initiated by the host, plant or microbiota. These include the KYN pathway (90–95% of Trp), serotonin pathway (1–2% of Trp) and indolic pathway (4–6% of Trp). Several compounds, including indoxyl sulfate (IS), kynurenine (KYN), kynurenic acid (KYNA), and indole-3-acetic acid (IAA), are recognized as uremic toxins and are marked with blue boxes. The compound acting as AhR ligands is marked with an asterisk (*)

The essential amino acid Trp is mainly degraded by three known metabolic pathways that can be initiated in the host, plant or microbiota: the KYN pathway (90–95% of Trp), serotonin pathway (1–2% of Trp) and indolic pathway (4%-6% of Trp) [19] (Fig. 2). In the KYN pathway, Trp is converted to N-formylkynurenine (NFK) by the rate-limiting enzymes tryptophan 2,3-dioxygenase (TDO) and indoleamine-2,3-dioxygenase (IDO-1/2). NFK is converted to KYN by kynurenine formamidase (AFMID). Subsequently, KYN is converted to 3-hydroxykynurenine (3-HK) by kynurenine 3-monooxygenase (KMO). Then, 3-HK is converted by kynureninase (KYNU) to 3-hydroxyanthralinic acid (3-HAA), which is converted by 3-hydroxyanthranilate 3,4-dioxygenase (HAAO) to quinolinic acid (QA). QA can be converted to NAD^+^, a key coenzyme in energy metabolism. 3-HK can also be catalyzed by kynurenine amino transferase (KAT) to produce xanthurenic acid (XA). KYN is also converted to anthralinic acid (AA) by KYNU. KAT can catalyze KYN to produce kynurenine quinolinic acid, also known as kynurenic acid (KYNA) [20]. TDO is highly expressed in the liver and brain, and IDO-1/2 is widely expressed in various tissues [2, 21].

In the serotonin pathway, Trp is metabolized by Trp hydroxylase enzyme (TpH), which produces 5-hydroxytryptophan (5-HTP). 5-HTP is further metabolized into 5-hydroxytryptamine (5-HT), also known as serotonin [22].

In the indole pathway, Trp is converted into indole by tryptophanase-positive microbiota. Indole is absorbed in the liver and then oxidized by cytochrome P450 family 2 subfamily E member 1 (CYP2E1) to hydroxyindole, which is converted into IS by sulfotransferases [23]. Some bacterial species use Trp and metabolize it to various indolic derivatives. For example, *Lactobacillus* spp. metabolize Trp to indole-3-aldehyde (I3A), *Bifdobacterium* spp. metabolize Trp to indole-3-lactic acid (ILA), and *Bacteroides* spp. metabolize Trp to indole-3-acetic acid (IAA) [8].

Trp photolysis by ultraviolet or visible light triggers several photochemical products, such as 1-(1H-indol-3-yl)-9H-pyrido[3,4-b]indole [24] and 6-formylindolo[3,2-b]carbazole (FICZ) [25].

Tryptophan-derived phytochemical indole-3-carbinol (I3C), which is produced in cruciferous brassica genus vegetables, including cauliflower, cabbage, and brussels sprouts, can be converted into indolo[3,2-b]carbazole (ICZ) by nonenzymatic condensation reactions in the stomach [26].

**Fig. 3:**
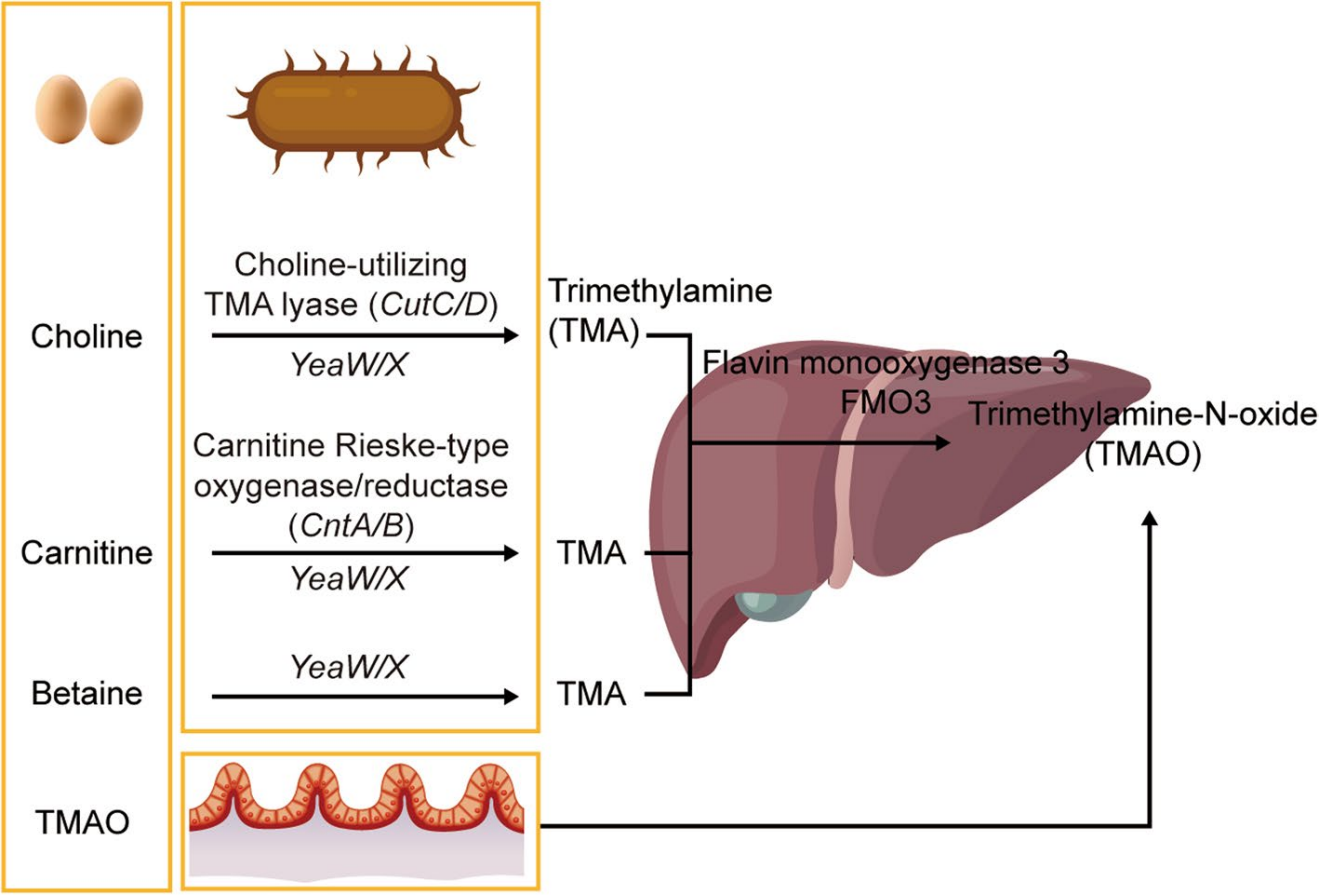
Pathways of trimethylamine-N-oxide (TMAO) production. Foods are enriched in TMAO precursors (choline, carnitine and betaine) or TMAO itself. Choline can be metabolized to trimethylamine (TMA) by the choline-utilizing TMA lyase (*CutC/D*). L-carnitine can be metabolized to TMA by the carnitine Rieske-type oxygenase/reductase (*CntA/B*). *YeaW* and *YeaX*, the homologs of *CntA/B*, can also metabolize choline, carnitine and betaine to generate TMA. The above processes occur in the microbiota. TMA produced in the gut is absorbed into the blood and transported to the liver, where flavin monooxygenase 3 (FMO3) catalyzes TMA into TMAO. Dietary TMAO can bypass processing by the gut microbiota before intestinal absorption

Choline is derived from eggs, fish and meat and can be metabolized to trimethylamine (TMA) by the choline-utilizing TMA lyase (*CutC/D*). L-carnitine is found in red meat and fish and can be metabolized to TMA by the carnitine Rieske-type oxygenase/reductase (*CntA/B*) [27–29]. *YeaW* and *YeaX*, the homologs of *CntA/B*, can also metabolize choline, carnitine and betaine to generate TMA. These effects are dependent on the gut microbiota [30]. TMA produced in the gut is absorbed into the blood and transported to the liver, where flavin monooxygenase 3 (FMO3) catalyzes TMA into TMAO [31]. Apart from dietary precursors of TMAO, most preformed TMAO, which is independent of gut microbes, is found in fish, humans [32] and rats [31, 33] (Fig. 3).

The European Uremic Toxin Work Group in 2003 classified uremic toxins into three categories based on dialysis clearance and physicochemical characteristics. The first category comprises small molecule toxins with a molecular weight of less than 500 Da, solubility in water, non-protein binding, and easy elimination through hemodialysis (HD), which include urea, creatinine, creatine, uric acid and xanthine. The second category comprises medium molecule toxins with a molecular weight exceeding 500 Da which are less efficiently cleared by HD; these toxins include β2-microglobulin, interleukin (IL)-1β, IL-6 and tumor necrosis factor (TNF)-α. Finally, protein-bound toxins are difficult to eliminate using conventional dialysis techniques, including PCS, IS, KYN, KYNA and IAA [34]. However, a conference on uremic toxins in 2020 challenges this classification as follows: First, the current physiochemical subdivisions based on molecular weight can be considered arbitrary and artificial. Second, the protein-bound degree of these uremic solutes is variable, and the molecular weights of these solutes remain uncertain. Third, the HD in the original classification only applies to conventional HD and not peritoneal or other dialysis. Fourth, the original classification does not consider the compartmental partitioning behavior of solutes within the body. Fifth, some uremic toxins already exist before the initiation of dialysis. Therefore, experts recommended that the definition of uremic toxins should be based on HD strategies, membranes, and removal patterns while adapting to technological advancements [35]. In addition, experts approved a scoring system in 2008 for classifying uremic toxins according to the experimental and clinical evidence of their toxicity. The highest-scoring uremic toxins were PCS, β2-microglobulin, asymmetric dimethyl arginine, KYN, carbamylated compounds, fibroblast growth factor (FGF)-23, IL-6, TNF-α and symmetric dimethyl arginine. The second highest-scoring uremic toxins are advanced glycation end products, IS, uric acid, ghrelin, IAA, parathyroid hormone, phenyl acetic acid, TMAO, retinol binding protein, endothelin, immunoglobulin light chains, IL-1β, IL-8, neuropeptide Y, lipids and lipoproteins [36]. Based on a new classification schema proposed by experts [35], this review further summarized the classification of uremic toxins according to metabolic pathways and dialysis modalities (Table 1).

**Table 1:** New classification of uremic toxins based on metabolic pathways and dialysis modalities [35]

Characteristics	Uremic toxin sources	Molecular weight	Dialysis modalities	Metabolic pathways and uremic toxin products
Protein-bound (Protein-bound ≥ 80%)	Exogenous (Gut-derived)	< 0.5 kDa	Low-flux HD; High-flux HD; High-flux HDF; Medium cutoff HDx; High cutoff HD;	Tryptophan metabolism (IS, KYN, IAA, KYNA [2]); Tyrosine metabolism (PCS [18]); Methionine metabolism (Homocysteine [173]); Maillard reaction (carboxymethyl lysine [174])
Water soluble (Protein-bound < 80%)	Exogenous and exogenous (Both gut-derived and endogenous metabolism)	< 0.5 kDa	Low-flux HD; High-flux HD; High-flux HDF; Medium cutoff HDx; High cutoff HD;	Choline, carnitine and betaine metabolism (TMAO [31]); Arginine methylation (asymmetric dimethylarginine, symmetric dimethylarginine [175]); Purine metabolism (uric acid [176]); Carbamylation (carbamylated compounds [177])
	Endogenous (endogenous metabolism)	0.5–15 kDa	High-flux HD; High-flux HDF; Medium cutoff HDx; High cutoff HD	Cytokine (IL-8); Structural protein (β_2_-microglobulin)
		> 15–25 kDa	High-flux HDF; Medium cutoff HDx; High cutoff HD	Cytokines (TNF, IL-18, IL-10, IL-6, FGF-2); Hormone (prolactin); Structural proteins (kappa-FLC, myoglobin, sTNFR2, complement factor D);
		> 25–58 kDa	Medium cutoff HDx; High cutoff HD	Cytokines (pentatraxin-3, FGF-23, CX3CL1, CXCL12, IL-2); Structural proteins (sTNFR1, lambda-FLC, YKL-40); Maillard reaction (advanced glycosylation end products [178])
		> 58 kDa	High cutoff HD	Modified albumin

*HD* hemodialysis, *HDF* hemodiafiltration, *HDx* expanded HD, *IS* indoxyl sulfate, *KYN* kynurenine, *IAA* indole-3-acetic acid, *KYNA* kynurenic acid, *PCS* p-cresyl sulfate, *TMAO* trimethylamine-N-oxide, *IL* interleukin, *TNF* tumor necrosis factor, *FGF* fibroblast growth factor, *sTNFR* soluble tumor necrosis factor receptor, *CX3CL* chemokine (C-X3-C motif) ligand, *YKL-40* chitinase-3-like protein 1

## AhR signaling

Compounds produced by Trp metabolism have been demonstrated to be potential AhR ligands, including KYN, KYNA, XA, 3-HK, 3-HAA, QA, tryptamine, IAA, 3-methylindole (skatole), I3A, ILA, indole, IS, I3C, ICZ, FICZ [19], 5-HTP [37], and indole-3-acetaldehyde (IAAld) [8]. IS is a potent ligand of AhR that exhibits 500-fold greater potency in the transcriptional activation of human AhR than mouse AhR [23]. FICZ has structural similarities to ICZ, and both are important endogenous AhR agonists. FICZ binds to the AhR with higher affinity than tetrachlorodibenzo-p-dioxin (TCDD), a well-known potent agonist of AhR [38]. However, the precursor I3C acts as a weak AhR ligand [39]. Under pathological stimuli, AhR is widely expressed in a variety of cells, including epithelial cells [40], vascular smooth muscle cells [41], endothelial cells, immune cells [42], hepatocytes [43], astrocytes and neurons [44].

**Fig. 4:**
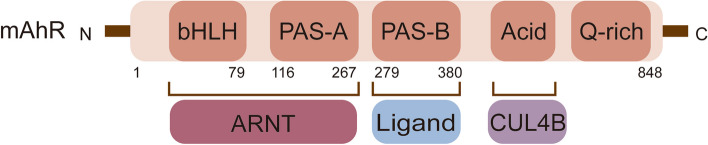
The structure of mouse AhR. AhR contains a basic helix-loop-helix (bHLH) domain and two repeats of the Per-Arnt-Sim (PAS) domain, known as PAS-A and PAS-B. The bHLH and PAS-A domains of AhR are responsible for ARNT binding, and the PAS-B domain is responsible for ligand binding. The C-terminal acidic domain of AhR interacts with cullin 4B (CUL4B)

AhR is a member of the bHLH-PAS family and is an evolutionarily conserved transcription factor. Structurally, AhR contains a bHLH domain and two repeats of a PAS domain, known as PAS-A and PAS-B [45–47] (Fig. 4). Under physiological conditions, the AhR PAS-B domain is attached to heat shock protein 90 (HSP90) [48]. The AhR bridge motif between PAS-A and PAS-B tightly binds to the HSP90 dimer and is threaded through the lumen of HSP90. HSP90 plays a crucial role in maintaining a high-affinity ligand-binding conformation. The amino acid residues connecting AhR PAS-B to the C-terminal transactivation domain form a long loop that folds back to the AhR PAS-B domain and interface with X-associated protein 2 (XAP2, also known as ARA9 or AIP), potentially interacting with the co-chaperone p23 [45]. These interactions effectively sequester the AhR molecule within the HSP90/XAP2/p23 complex, thereby stabilizing AhR in the cytoplasm [48]. In the presence of ligands, the DE-loop and a group of conserved pocket inner residues within the AhR PAS-B domain are responsible for ligand binding [48]. Activation of AhR involves conformational changes that expose the nuclear localization sequence in its N-terminal region, triggering translocation to the nucleus. In the nucleus, this complex dissociates and releases AhR [49]. Subsequently, AhR binds to the aryl hydrocarbon receptor nuclear translocator (ARNT) through interactions in the bHLH and PAS-A domains [50]. The outcome is the recruitment of transcriptional coactivators, such as histone acetyltransferase steroid receptor coactivator (SRC)-1, SRC-2 and p300, IκB kinase α (IKKα), brahma-related gene 1, and RNA initiation factors, to target promoters to enhance transcriptional activity [51–53]. This AhR/ARNT/coactivator complex binds to target genes containing consensus DRE or XRE (referred to as dioxin-response element or xenobiotic-responsive element) sequences (5’-GCGTG-3’) and regulates the transcription of target genes, including *Cyp1a1*, *Cyp1a2* [53], aryl hydrocarbon receptor repressor (*AhRR*) [54], nucleotide-binding oligomerization domain, leucine rich repeat and pyrin domain containing 3 (*Nlrp3*) [55], *IL-10* [9] and *IL-22* [56]. Additionally, AhR regulates the transcription of target genes that do not harbor the canonical XRE recognition site in promoter regions by interacting with additional transcription factors, such as estrogen receptor (ER), krüppel-like factor 6 (KLF6), nuclear factor-κB (NF-κB), and MAF bZIP transcription factor (c-Maf) [57–60]. Furthermore, AhR can directly regulate the transcription of nuclear factor erythroid 2-related factor 2 (*Nrf2*) [61].

**Fig. 5:**
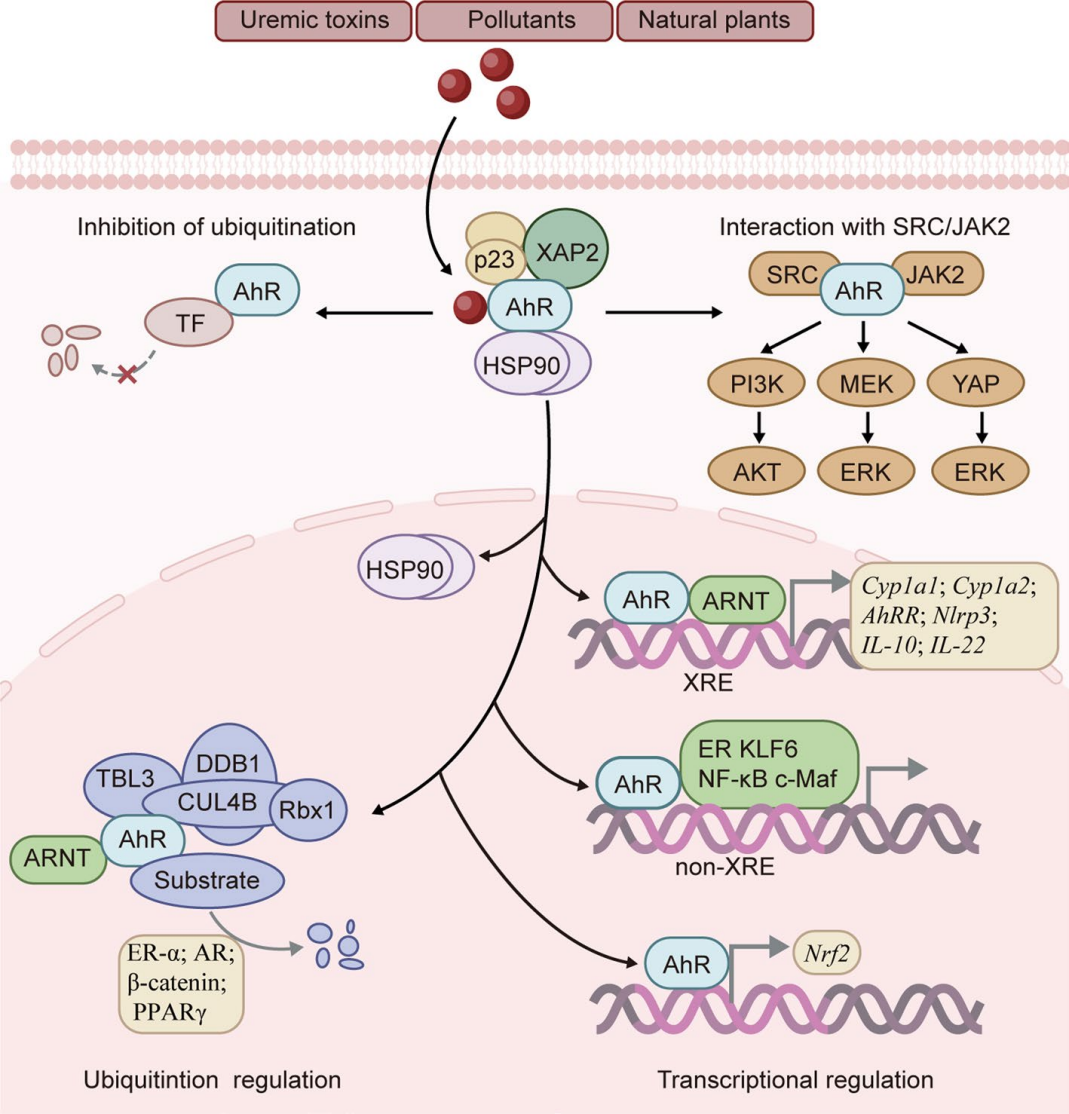
AhR signaling pathway. Before ligand binding, AhR remains stable in the cytoplasm within the HSP90/XAP2/p23 complex. When exposed to AhR ligands, such as uremic toxins, pollutants or natural plants, AhR changes its conformation, thus exposing the nuclear localization sequence in its N-terminal region and triggering translocation to the nucleus. In the nucleus, AhR is released from this complex and activated. Activated AhR binds to ARNT and some coactivators to regulate the transcription of target genes containing consensus XRE (xenobiotic response element), such as *Cyp1a1*, *Cyp1a2*, *AhRR*, *Nlrp3*, *IL-10* and *IL-22*. In addition, AhR regulates the transcription of target genes that do not harbor the canonical XRE recognition site in their promoter regions by interacting with additional transcription factors, such as ER, KLF6, NF-κB and c-Maf. Furthermore, AhR directly regulates the transcription of *Nrf2*. Additionally, AhR, together with DDB1, Rbx1, TBL3, ARNT and CUL4B, assembles into the novel CUL4B ubiquitin ligase complex CUL4B^AhR^ to regulate target proteins for ubiquitin degradation, such as ER-α, AR, β-catenin and PPARγ. Ligand-activated cytoplasmic AhR can act as a protein adaptor that links SRC to JAK2, activating the PI3K/AKT, MEK/ERK and YAP/ERK signaling pathways. AhR can also protect tissue factor (TF) from ubiquitination and degradation

The transcriptional activity of AhR cannot explain all the cellular functions attributed to this receptor. Several studies have reported that AhR also functions as an E3 ubiquitin ligase. In the nucleus, AhR, together with damaged-DNA binding protein 1 (DDB1), RING-box protein 1 (Rbx1), transducin-β-like protein 3 (TBL3), ARNT and scaffold protein cullin 4B (CUL4B), forms a novel CUL4B ubiquitin ligase complex, CUL4B^AhR^. Ligand-activated AhR acts as a substrate-specific adaptor component targeting ER-α and androgen receptor (AR) for ubiquitin-mediated degradation. Furthermore, a study confirmed that the conserved C-terminal acidic domain of AhR interacts with the N-terminal region of CUL4B [62]. The role of AhR E3 ubiquitin ligase was implicated in β-catenin degradation, which occurs independently but cooperatively with the APC-dependent pathway to suppress intestinal carcinogenesis [63]. AhR also targeted peroxisome proliferator-activated receptor γ (PPARγ) for proteasomal degradation to regulate adipocyte differentiation [64]. However, the specific mechanism by which this molecular switch mediates the transcriptional activity or E3 ubiquitin ligase activity of AhR has not been fully elucidated. Several investigations have attempted to address this question, with Luecke-Johansson et al. proposing that ARNT plays a crucial role in determining the dual functions of AhR. Their findings revealed that the absence of ARNT significantly impeded the transcriptional activation of AhR but did not affect its E3 ubiquitin ligase function [65]. Kuocheng Lu et al. also demonstrated that different IS concentrations can modulate ARNT as a molecular switch for AhR. Low-dose IS exposure increased nuclear ARNT expression, facilitating the formation of the IS/AhR/ARNT complex in the nucleus. However, high-dose IS exposure decreased ARNT expression, inhibiting the transcriptional activity of AhR in the nucleus and increasing the function of AhR E3 ligase in the cytoplasm [66]. However, it should be noted that the two aforementioned studies ignored the involvement of ARNT in CUL4B^AhR^ complex formation. The cytoplasmic functions of AhR have been gradually elucidated. Ligand-activated cytoplasmic AhR has been reported to act as a protein adaptor that links SRC to janus kinase 2 (JAK2) and mediates SRC phosphorylation by JAK2, which activates the phosphatidylinositol 3-kinase (PI3K)/AKT, mitogen-activated extracellular signal-regulated kinase (MEK)/extracellular signal-regulated kinase (ERK) [67] and yes-associated protein (YAP)-ERK signaling pathways [68]. Ligand-activated cytoplasmic AhR also protects tissue factor (TF) from ubiquitination and degradation to increase thrombotic risk [41] (Fig. 5).

The AhR signaling pathway is regulated at three levels: (i) the production and metabolism of ligands that act as agonists or antagonists [8] and (ii) activity disruption by competitors such as AhRR and hypoxia inducible factor (HIF)-1α. AhRR inhibits AhR signal transduction by binding to XREs and ARNT [69] or recruiting corepressors such as ankyrin repeat and LEM domain containing 2 gene, histone deacetylase (HDAC) 4 and HDAC5, which form a negative feedback loop to prevent the overactivation of AhR [70]. HIF1α inhibits AhR activity by interacting with ARNT (also known as HIF-1β) [71]. (iii) Degradation of AhR. After AhR is separated from DNA, it is exported from the nucleus and subjected to proteasomal degradation [72]. AhR can be phosphorylated in a glycogen synthase kinase-3-dependent manner, leading to lysosomal degradation of the AhR protein [73]. Conversely, AhR can be deubiquitinated by the deubiquitinating enzyme ubiquitin C-terminal hydrolase L3 [74]. These mechanisms ensure the proper balance of AhR biology.

## Uremic toxin-activated AhR in kidney diseases

Proximal renal tubular epithelial cells (RTECs), which possess various transporters on the cell membrane, are responsible for the absorption and secretion of substances, including drugs, metabolites and environmental toxins [75]. Most uremic toxins are transported via the solute carrier family members organic anion transporters 1 and 3 (OAT1 and OAT3) [75]. A study revealed elevated levels of plasma uremic toxins such as IS, XA and KYN in OAT1 knockout mice [76]. An imbalance between the production and excretion of uremic toxins can contribute to their accumulation within the body, which disturbs normal physiological functions and energy metabolism [1]. When renal function is impaired, the accumulation of uremic toxins accelerates the progression of kidney diseases by activating the AhR signaling pathway, and this damage occurs not only in the kidney but also in other organs, such as the heart, vessel, liver and muscle (Table 2).

**Table 2:** The impact of AhR activation on kidney diseases and complications

Diseases	AhR-expressing cell types	Biological effects	Signaling pathways	References
CKD	Human aortic vascular smooth muscle cell	Accelerate thrombosis	AhR stabilized TF expression by inhibiting TF ubiquitination and degradation.	[41]
	HUVEC and PBMC	Accelerate atherogenesis	AhR increased TF expression.	[42]
	HUVEC	Accelerate thrombosis	Increased TF expression was regulated by AhR/p38 MAPK/NF-κB pathway.	[93]
	HUVEC	N/A	AhR promoted neuronal pentraxin 1 transcription.	[94]
	Human dermal microvascular endothelial cell	Suppress postischemic angiogenesis and promote PAD	AhR augmented β-catenin ubiquitination and degradation and then suppressed Wnt/β-catenin signaling pathway.	[98]
	Skeletal muscle cell	Exacerbate the ischemic myopathy and PAD	N/A	[99]
	HUVEC	Exacerbate vascular inflammation	AhR stimulated the transcriptional activity of activator protein 1 and then upregulated E-selectin expression, leading to the aggravation of leukocyte recruitment to the vascular wall.	[101]
	Macrophage	Promote inflammation	AhR increased the transcription of *Socs2* and *Tnf-α.*	[102]
	N/A	Promote blood–brain barrier disruption associated with cognitive impairment	N/A	[87]
	Astrocyte	Induce anxiety, cognitive impairment, astrocyte reactivation and neuronal activity enhancement	AhR downregulated GLT1 expression and activity and promoted pro-oxidant NOX1 expression.	[44]
	RTEC	Enhance IS clearance	Elevated IS levels induced robust increases in the expression and transport activity of OAT1 by activating the AhR/ARNT and EGFR pathways.	[40]
	Hepatocyte	Increase hepatic cyclosporine clearance	AhR upregulated P-glycoprotein expression and activity.	[43]
DN	MC and RTEC	Promote MC activation and extracellular matrix production	AhR was bound to the promoters of *Cox-2*, fibronectin, and collagen IV.	[122]
AKI	RTEC	Inhibit renal inflammation, pathological injury and apoptosis	AhR inhibited NF-κB and JNK pathways.	[128]
	RTEC	Promote tubular cell survival against cisplatin toxicity and protect the kidney from cisplatin-induced acute injury	Elevated miR-125b transcriptionally by Nrf2 inhibited AhRR, increasing the transcriptional activity of AhR, promoting MDM2 expression, and then inhibiting p53 activity.	[130]
	N/A	Limit renal damage during malaria	N/A	[131]
	RTEC	Promote apoptosis and renal damage	AhR induced oxidative stress by increasing ROS.	[132]
	RTEC	Accelerate cellular senescence, kidney dysfunction and tubular injury	AhR upregulated EZH2 expression, and EZH2 conversely enhanced AhR expression via weakening H3K27me3 transcriptional inhibition on the AhR promoter.	[133]
	RTEC	Do not affect cellular senescence	N/A	[134]
OSA	N/A	Promote the progression of HTN induced by CIH	AhR antagonist CH223191 prevented the increase in systolic blood pressure by 53 ± 12% and diastolic blood pressure by 44 ± 16%.	[136]

*AhR* aryl hydrocarbon receptor, *CKD* chronic kidney disease, *DN* diabetic nephropathy, *AKI* acute kidney injury, *OSA* obstructive sleep apnea, *HUVEC* human umbilical vein endothelial cell, *PBMC* peripheral blood mononuclear cell, *TF* tissue factor, *PAD* peripheral artery disease, *MAPK* mitogen-activated protein kinase, *NF-κB* nuclear factor kappa-B, *ARNT* aryl hydrocarbon receptor nuclear translocator, *Socs2* suppressor of cytokine signaling 2, *Tnf* tumor necrosis factor, *GLT1* glutamate transporter 1, *NOX1* NADPH oxidase 1, *RTEC* renal tubular epithelial cell, *IS* indoxyl sulfate, *EGFR* epidermal growth factor receptor, *OAT1* organic anion transporter 1, *MC* mesangial cell, *Cox2* cyclooxygenase 2, *JNK* c-Jun N-terminal kinase, *AhRR* aryl hydrocarbon receptor repressor, *MDM2* mouse double minute 2, *miR* miroRNA, *Nrf2* nuclear factor erythroid 2-related factor, *ROS* reactive oxygen, *EZH2* enhancer of zeste homolog 2, *CIH* chronic intermittent hypoxia, *HTN* hypertension

### Uremic toxin-activated AhR in CKD

CKD is defined by persistent urine abnormalities and structural or functional impairments suggestive of a loss of functional nephrons [77]. CKD is a major public health problem that affects nearly 9.1% of the global population [78]. As the global population ages and the incidences of diabetes, hypertension and other diseases increase, the incidence of CKD also gradually increases [77]. The majority of patients with CKD are at high risk of cardiovascular disease (CVD) and death. When patients with CKD progress to end-stage renal disease (ESRD), the optimal treatment strategy is renal replacement therapy, such as dialysis or kidney transplantation, which has limited accessibility and is extremely susceptible to cardiovascular mortality [77]. It was estimated that CVD mortality in patients who underwent kidney transplantation is 2.3 times greater than that in the general population [79].

#### The accumulation of uremic toxins and activation of AhR in CKD

In CKD patients, plasma Trp levels were unchanged, and metabolites of Trp, including KYN, 5-HTP, serotonin and QA, were significantly increased at CKD stage 3 [80]. Plasma KYNA [80], IAA [81] and serum IS [82] levels increased significantly at the CKD stage 4. These uremic toxins increased progressively with increasing CKD stage [80–82]. IAA levels decreased substantially after kidney transplantation. Nontransplanted CKD patients with above-median IAA concentrations had a significantly higher risk of overall mortality and cardiovascular events than patients with below-median levels [81]. IS is also an independent risk factor for cardiovascular events in patients with CKD [82, 83]. IS has been shown to be positively correlated with aortic calcification and pulse wave velocity [82]. Patients on HD with high plasma IS concentrations were at a higher risk of developing first heart failure [84]. PCS is another widely studied and protein-bound uremic toxin. Serum PCS levels were increased in CKD patients and associated with CKD progression and all-cause mortality [85]. Similarly, the levels of serum uremic toxins have been observed in animal models of CKD. The serum IS concentrations were significantly higher in adenine diet-fed mice and rats, IS mice given water containing IS, and 5/6 nephrectomized rats than in controls, and the serum KYN levels were elevated in adenine diet-fed mice [86, 87]. Plasma TMAO was markedly increased, and elevated TMAO was associated with a 2.8-fold increase in the risk of 5-year all-cause mortality in CKD patients. High TMAO levels portend poorer prognosis among non-CKD subjects [88]. Taken together, these studies indicate that some uremic toxins are independent predictors of overall mortality, CKD progression and cardiovascular events in CKD patients.

With the accumulation of AhR ligands in serum, upregulated AhR expression and activation are also observed in CKD patients and animals. AhR activity was higher in the sera of 20 ESRD patients on HD than in those of controls (activity range 3.02–7.62 vs. 1.1–2.38) [41]. Similarly, another clinical study involving 116 patients with CKD revealed a significant increase in serum AhR activity. The mRNA levels of AhR target genes *Cyp1a1* and *AhRR* were increased in patient blood cells, suggesting activation of the AhR signaling pathway in CKD patients. In addition, significant increases in serum AhR activity and *Cyp1a1* mRNA level in the aorta and heart were detected in both 5/6 nephrectomy-induced CKD mice and mice injected with IS for 5 consecutive days, whereas increased *Cyp1a1* mRNA level was not observed in AhR knockout mice [89]. In kidneys of mice with unilateral ureteral obstruction-induced renal fibrosis, an increase in AhR mRNA level was accompanied by significant increases in the expressions of AhR target genes, including *Cyp1a1*, *Cyp1a2* and *Cyp1b1*, suggesting AhR signaling pathway activation in mouse kidneys [90]. AhR activation was also confirmed in the kidneys of 5/6 nephrectomized rats and patients with idiopathic membranous nephropathy and IgA nephropathy [91].

The above studies consistently suggest that uremic toxins are accumulated and AhR is activated during the progression of CKD.

#### The function of uremic toxin-activated AhR in CKD

It was reported that IS and uremic serum induced AhR activation, as validated by nuclear translocation and increased expressions of target genes, including *Cyp1a1, Cyp1b1* and *AhRR,* which were abrogated by AhR antagonists CB7993113 and CH223191 [41]. Approximately 90% of IS circulates in a protein-bound way among HD patients [92]. A study indicated that both albumin-bound and free IS induced dose-dependent AhR activity in vascular smooth muscle cells [41]. Numerous studies have focused on the harmful effects of AhR activation by uremic toxins on cardiovascular dysfunction during CKD. Serum IS levels in ESRD patients were positively correlated with serum AhR activity and vascular smooth muscle cellular TF activity. In primary cultured human aortic vascular smooth muscle cells, the activation of AhR by IS stabilized TF via inhibiting TF ubiquitination and degradation, thus accelerating thrombosis [41]. Similar studies also showed that IS and IAA upregulated TF expression by activating AhR in human umbilical vein endothelial cells (HUVECs) and peripheral blood mononuclear cells. And the effect was suppressed by treatment with AhR siRNA or the AhR inhibitor geldanamycin. Plasma TF levels were significantly higher in CKD patients than in healthy controls, and TF levels were even higher in CKD patients requiring HD than in non-dialysis patients. In addition, plasma TF levels were positively correlated with IS and IAA levels. The procoagulant state induced by increased TF expression and the direct proatherogenic effect of AhR activation accelerated atherogenesis in CKD [42]. However, other researchers observed that IAA-activated AhR promoted TF transcription independently of binding to the TF promoter in HUVECs. In fact, TF upregulation by IAA was regulated by the AhR/p38 mitogen-activated protein kinase (MAPK)/NF-κB pathway, which increased thrombotic risk [93]. Activated AhR by uremic solutes IS and IAA, as well as TCDD and FICZ, promoted neuronal pentraxin 1 transcription in HUVECs, and the mRNA level of neuronal pentraxin 1 was increased in the aortas of adenine-induced CKD mice [94]. Another study showed that IS reduced the fast transient outward potassium current-related proteins and current densities by activating the reactive oxygen (ROS)/MAPK and NF-κB signaling pathways, prolonging action potential duration and QT interval in neonatal rat ventricular myocytes and hearts of CKD rats. This result helps to account for the high prevalence of ventricular arrhythmias related to sudden cardiac death in CKD patients [95].

CKD is well recognized as a distinct contributor to cardiac hypertrophy. A study clarified the relationship between uremic toxins and cardiac hypertrophy. Treatment of cardiomyocytes with uremic serum collected from patients with CKD stage 5 who have accumulated diverse uremic toxins induced mitochondrial oxidative damage. Mitochondrial damage increased VDAC-mediated mitochondrial outer membrane permeabilization, leading to the release of mitochondrial DNA. Mitochondrial DNA activated cyclic GMP-AMP synthase/stimulator of interferon gene/NF-κB pathway and then stimulated ornithine decarboxylase upregulation and putrescine accumulation, which promoted cardiac hypertrophy [96].

CKD imposes a potent and independent risk for peripheral artery disease (PAD). In a study involving a cohort of 1,091,201 patients, those with CKD exhibited a striking threefold increase in the prevalence of PAD compared with the non-CKD patients [97]. A recent study demonstrated that plasma IS levels were elevated by 1.6-fold, KYN levels were raised by 2.2-fold, and KYNA and XA levels were heightened by 1.5-fold in PAD patients with adverse limb events compared to those without adverse limb events. However, there were no significant differences in the levels of Trp, AA, or QA between the 2 groups. Plasma from PAD patients with adverse events activated AhR activity in human dermal microvascular endothelial cells 60% more compared with the group without adverse events. Uremic toxins were found to suppress the Wnt/β-catenin pathway by augmenting AhR-mediated β-catenin ubiquitination and degradation in human dermal microvascular endothelial cells, which was also verified in adenine-induced CKD and IS solute-specific mouse models with hindlimb ischemia. Notably, inhibiting AhR activity with CH223191 normalized postischemic angiogenesis in adenine-induced CKD mice to a non-CKD level [98]. Another study explored the role of AhR activation in the myopathy of PAD and CKD. The expression and activity of AhR in skeletal muscle were greater in PAD patients with CKD than in PAD patients with normal renal function or non-PAD adult controls. Skeletal muscle-specific AhR knockout promoted ischemic muscle perfusion recovery and arteriogenesis and preserved ischemic muscle mass, contractile function, mitochondrial respiratory function and paracrine vasculogenic signaling between myofibers and vascular cells in adenine-induced CKD mice with hindlimb ischemia. These findings indicate that AhR inhibition is a potential therapeutic for PAD patients with CKD [99]. These studies implicate that retained uremic solutes in CKD patients drive PAD progression by disrupting angiogenesis and muscle health in an AhR-dependent manner.

Increasing reports show that uremic toxin-activated AhR creates a vicious cycle between oxidative stress and inflammation, which aggravates the chronic inflammatory environment in CKD. It has been reported that IS induces ROS production [95]. IS-upregulated ROS promoted the expressions of cAMP response element-binding protein and NF-κB, increasing NADPH oxidase (NOX) 4 expression, an enzyme catalyzing the reduction of molecular oxygen to ROS in proximal renal tubules [100]. IS-induced ROS production led to c-Jun N-terminal kinase (JNK) and NF-кB activation independent of AhR regulation in HUVECs. This study also showed that IS-induced AhR activation stimulated the transcriptional activity of activator protein 1 and subsequently upregulated E-selectin expression in HUVECs, which led to the aggravation of leukocyte recruitment to the vascular wall and vascular inflammation. Endothelial cell-specific AhR knockout inhibited leukocyte recruitment [101]. Crosstalk between AhR and NF-κB is also observed in macrophages. During the early stages of IS stimulation, IS-activated AhR was associated with the NF-κB p65 subunit, leading to mutual inhibition of AhR and NF-κB in the cytoplasm. Subsequently, IS-activated AhR translocated into the nucleus and promoted the transcription of suppressor of cytokine signaling 2 (*Socs2*), a negative modulator of NF-κB, thus inhibiting NF-κB signaling pathway activation. Finally, the mutual inhibition of AhR and NF-κB was diminished, and free activated AhR induced TNF-α expression by binding to the promoter of *Tnf-α* [102]. Both free and albumin-bound IS triggered proinflammatory macrophage activation and the expression of proinflammatory cytokines, such as IL-1β, TNF-α and monocyte chemotactic protein 1, in 5/6 nephrectomy-induced CKD mice [103]. These findings indicate that AhR may promote inflammation in CKD.

Furthermore, uremic toxins impair the antioxidant capacity of cells against oxidative stress. Glutathione is a marker of oxidative stress and is known as the most potent antioxidant [104]. A study showed that IS, phenyl sulfate, and PCS, but not IAA, at CKD concentrations led to decreases in total glutathione levels, thus rendering tubular epithelial cells vulnerable to oxidative stress [105].

Recently, researchers have realized that CKD patients have a higher risk of developing cognitive impairment and dementia, even in the early stage of CKD [106]. It is noted that the accumulation of uremic toxins may harm cerebral endothelium and cognitive function in CKD [107, 108]. Notably, serum free IS concentrations, but not PCS, were associated with lower cognitive function in patients with HD [109]. The effect of uremic toxins was experimentally explored, and an increase in serum IS concentrations was shown to promote blood–brain barrier disruption associated with cognitive impairment by AhR activation in CKD rats established by an adenine-rich diet or by 5/6 nephrectomy [87]. Similarly, 5/6 nephrectomy-induced CKD mice showed increased IS concentrations in both the blood and brain and AhR activation in the anterior cortex. CKD-induced anxiety, cognitive impairment, astrocyte reactivation in the anterior cingulate cortex, and neuronal activity enhancement in the anterior cingulate cortex and hippocampal CA1 neurons were ameliorated after knocking out neural lineage-specific and astrocyte-specific AhR or treating with AhR antagonist CH223191. Mechanistically, IS-activated AhR downregulated glutamate transporter 1 (GLT1) expression and activity and promoted pro-oxidant NOX1 expression in astrocytes, leading to enhanced neuronal activity and synaptotoxicity in the brain. The study indicates that astrocytic AhR promotes CKD-induced neuron-astrocyte interaction dysfunction and mental disorders [44].

Renal fibrosis is the common ultimate pathological feature of CKD. Uremic toxins are considered to play a determinant pathological role in the progression of renal fibrosis. Peripheral fibroblast activation and tubular injury are the hallmarks of renal fibrosis [110]. In fibroblasts, IS accumulation promoted renal fibroblast activation via an HSP90-dependent pathway [110]. In proximal RTECs, IS and PCS significantly activated the intrarenal renin–angiotensin–aldosterone system by increasing renin, angiotensinogen and angiotensin 1 receptor expressions, and decreasing angiotensin 2 receptor expression. IS and PCS also increased transforming growth factor β1 (TGFβ1) expression and activated the TGFβ/Smad pathway. IS and PCS induced the epithelial-mesenchymal transition (EMT) phenotype by increasing snail family transcriptional repressor expression. EMT was implicated in renal fibrosis [111]. Cellular senescence is a stress-induced cell cycle arrest independent of age. Senescent cells obtain increased secretion of cytokines, chemokines, growth factors, and proteases, which is referred to as the senescence-associated secretory phenotype [112]. Cellular senescence has been found in multiple kidney diseases, especially in CKD. Young CKD patients frequently exhibit characteristics of premature aging, including vascular aging, bone disease, muscle wasting, cognitive dysfunction and frailty. Chronic renal injury induces cellular senescence, and cellular senescence can also accelerate the progression of renal fibrosis [113]. Recent findings have revealed that uremic toxins mediate cellular senescence in CKD. IS and PCS decreased Klotho expression by enhancing DNA methylation of the Klotho gene in RTECs, thus promoting renal fibrosis [114]. IS can also induce the downregulation of Klotho expression and the production of proinflammatory cytokines in macrophages by stimulating M1 polarization. Overexpression of Klotho alleviated kidney fibrosis by inactivating NF-kB signaling and promoting macrophage M2 polarization [115].

An increase in body uremic toxins triggers remote metabolite sensing to mediate toxins and drug clearance. Membrane transporters are generally involved in metabolite sensing and are widely expressed in epithelial barriers. In proximal RTECs, elevated IS levels induced robust increases in the expression and transport activity of OAT1 by activating the AhR/ARNT and EGFR pathways, enhancing IS clearance. EGFR played a pivotal role in ARNT nuclear translocation, suggesting that crosstalk occurs between EGFR and AhR in IS sensing and signaling [40]. Additionally, IS increased the expression and activity of hepatocellular efflux transport protein P-glycoprotein (P-gp) during CKD by activating AhR, thus promoting the clearance of cyclosporine, a P-gp substrate, from the liver [43]. These results indicate that activated AhR promotes the detoxification process by upregulating the expression of membrane transporters in response to the uremic toxin IS during CKD. Unfortunately, increasing the expression of transporters may alter the clearance of drugs and produce secondary effects.

Several studies have observed renal and hepatic changes in systemic AhR knockout rats. AhR knockout rats exhibited urologic pathological changes such as bilateral renal and ureter dilation (hydronephrosis and hydroureter), as well as secondary medullary tubular and uroepithelial degenerative changes. However, AhR knockout mice exhibited impaired liver function, patent hepatic ductus venosus, and persistent hyaloid arteries in the eye [116]. These changes suggest that AhR plays significantly different roles in tissue development and body homeostasis in different species. Activated AhR is predominantly expressed in the proximal and distal tubules and periglomerular regions in animal models of CKD [86]. However, few studies have explored the role of uremic toxin-activated AhR in the renal tubular epithelium during CKD.

### Uremic toxin-activated AhR in DN

DN is defined as kidney damage due to diabetes and has become the predominant contributing factor to CKD. DN occurs in approximately 40% of people with type 2 diabetes (T2D) and type 1 diabetes (T1D) [117]. DN mainly manifests as hyperfiltration, urinary protein, and progressive decline in renal function [118].

#### The accumulation of uremic toxins and activation of AhR in DN

Notably, compared with nondiabetic patients, the plasma of diabetic patients had lower Trp levels and significantly higher Trp metabolite levels such as 5-HTP, 5-hydroxyindoleacetic acid, KYNA, 3-HK, and XA [119]. Serum IS levels were fourfold higher in streptozotocin (STZ)-induced DN mice compared with controls [120].

AhR expression is increased in DN patients [91]. An increase in AhR activity is also observed in DN patients. Serum AhR activity was increased in the microalbuminuria, macroalbuminuria and ESRD patients compared with normoalbuminuria subjects, and the ESRD group showed higher AhR activity compared with the microalbuminuria and macroalbuminuria groups. Moreover, the serum AhR activity was negatively correlated with eGFR and positively correlated with serum creatinine levels. These findings suggested that serum AhR activity is a significant independent risk factor for DN [121].

#### The function of uremic toxin-activated AhR in DN

One study confirmed the role and mechanism of AhR activation in DN. STZ-induced diabetic mouse kidneys exhibited elevation in glomerular mesangial cell (MC) activation, macrophage infiltration, extracellular matrix protein deposition, cyclooxygenase (COX-2)/prostaglandin E2 production, lipid peroxidation, oxidative stress, NOX activity and N-ɛ-carboxymethyl lysine formation, which was attenuated by AhR knockout or AhR inhibitor α-NF. N-ɛ-carboxymethyl lysine triggered the transportation of AhR to the nucleus, where it bound to the promoters of *Cox-2*, fibronectin and collagen IV to produce extracellular matrix proteins in MCs and RTECs. These results suggest that activated AhR plays an important role in MC activation, macrophage infiltration, and extracellular matrix protein accumulation in DN [122]. In addition, treatment with Tangshen Formula, a traditional Chinese herbal medicine, for 12 weeks, significantly attenuated inflammation, renal histologic injury and urinary albumin excretion by inhibiting the upregulation of AhR expression in DN rats [120].

### Uremic toxin-activated AhR in AKI

AKI is defined as the sudden loss of kidney function. Slow deterioration of kidney function or persistent kidney dysfunction in AKI is associated with irreversible loss of renal cells and nephrons, potentially leading to CKD [123]. The incidence of AKI is growing by 10% annually, and AKI affects up to 20% of hospitalized patients, with up to 50% of intensive care unit-admitted patients [124]. The main features of AKI induced by ischemia reperfusion (IR), drugs, and sepsis are apoptosis, oxidative stress, inflammation, mitochondrial dysfunction, and abnormalities within the renal vascular system [125, 126].

#### The accumulation of uremic toxins and activation of AhR in AKI

Clinical studies have shown that serum IS levels are significantly upregulated in AKI patients [127]. IR-induced AKI mice exhibited elevated plasma IS concentrations but no significant change in KYN. Renal AhR activity was increased in IR-induced AKI mice [86].

#### The function of uremic toxin-activated AhR in AKI

Studies showed that renal AhR expression was decreased in IR mice, along with impaired renal function, increased secretion of inflammatory factors and increased apoptosis. Treatment with the AhR agonist FICZ attenuated renal inflammation, pathological injury and apoptosis by inhibiting the NF-κB and JNK signaling pathways [128]. A similar study also revealed that IR-induced AKI mice treated with the AhR agonist leflunomide exhibited less apoptosis and necrosis and higher mitochondrial membrane potential than AKI mice. Leflunomide affected the infiltration of immune cells and stem cells in injured kidneys by increasing regulatory T cells, IL-10-positive cells and stem cell subsets (e.g., mesenchymal and hematopoietic stem cells and endothelial progenitor cells) and reducing IL-17- and IL-23-expressing cells [129]. In addition, activated AhR can relieve cisplatin-induced AKI. Elevated miR-125b transcription induced by Nrf2 inhibited the translation of AhRR mRNA, which increased the transcriptional activity of AhR. Activated AhR promoted the expression of mouse double minute 2 (MDM2), leading to the inhibition of p53 activity. The decrease in p53 promoted tubular cell survival against cisplatin toxicity and protected the kidney from cisplatin-induced acute injury [130]. AhR knockout mice were more susceptible to malaria and developed high plasma heme levels and AKI during malaria, suggesting that AhR limits renal damage during malaria [131]. These studies indicate that AhR may represent a novel renoprotective mechanism for AKI. However, the role of AhR in AKI remains controversial. Several studies have shown the pro-injury effect of AhR in AKI. AhR expression was increased in RTECs after cisplatin treatment. Knockdown of AhR by siRNA inhibited the IS-induced increase in ROS levels in cisplatin-treated RTECs, indicating that the IS/AhR/ROS axis contributes to oxidative stress. ROS elevation may result in apoptosis and renal damage in cisplatin-induced AKI [132]. A similar study showed that AhR was increased in cisplatin-induced AKI mice kidneys and RTECs. AhR inhibition by BAY2416964 and tubular conditional deletion of AhR both alleviated cisplatin-induced kidney dysfunction and tubular injury by inhibiting cellular senescence. Mechanistically, AhR upregulated the expression of methyltransferase enhancer of zeste homolog 2 (EZH2), and EZH2 conversely enhanced AhR expression via weakening H3K27me3 transcriptional inhibition on the AhR promoter [133]. This finding suggests that increased AhR is implicated in cisplatin-associated cellular senescence, and inhibition of AhR is a promising therapeutic strategy against AKI. Interestingly, a study showed that AhR was activated under anoxia or reoxygenation in primary proximal RTECs. The AhR inhibitor CH223191 did not affect cellular senescence under anoxia or reoxygenation [134].

### Role of AhR in other kidney-related diseases

Renal damage is one of the typical clinical manifestations of SLE. A study showed that AhR was significantly increased in B cells of SLE patients with renal injury compared to SLE patients without renal injury, indicating that AhR may be a potential marker for predicting SLE with renal damage [135].

Obstructive sleep apnea (OSA) is a highly prevalent sleep-related breathing disorder. The main hallmark of OSA is chronic intermittent hypoxia (CIH), which contributes to systemic hypertension (HTN). The CIH-induced HTN rat kidney cortex and medulla showed higher expression and activation of AhR. In CIH-induced HTN rats, administration of AhR antagonist CH223191 (5 mg/kg/day, gavage, daily) for 14 days prevented the increase in systolic blood pressure by 53 ± 12% and diastolic blood pressure by 44 ± 16%. These findings suggest that renal AhR activation promotes the progression of HTN induced by CIH [136].

## Therapeutic strategy

The accumulation of uremic toxins contributes to multiple organ injuries by activating AhR. Two principal therapeutic options are available to alleviate uremic toxin-induced injury: reducing the levels of uremic toxins and developing pharmacologic approaches to target AhR to mitigate their toxic effects [10] (Table 3).

**Table 3:** Therapeutic strategies for reducing uremic toxins or modulating AhR activation in kidney diseases

Therapies	Techniques	Principles	Functions	References
Blood Purification	Conventional HD	Diffusion	Removal of water-soluble small molecular-weight uremic toxins	[137]
	HDF	Diffusion and convection	Removal of small and middle molecular-weight uremic toxins	[137]
	Hemoperfusion	Adsorption	Removal of middle and large molecular-weight and protein-bound uremic toxins	[137–141]
Gastrointestinal dialysis	Carbon adsorbent AST-120	The intestinal absorption and subsequent fecal excretion of uremic toxin precursors	AST-120 reduced renal and serum uremic toxins and attenuated neointima formation in CKD mice. But it did not attenuate renal injury in CKD mice and slow disease progression in CKD patients.	[142–145]
Nutritional therapy	Low protein diet	Reduction of substrate intake for uremic toxin generation	A low protein diet showed lower plasma and urinary uremic toxin levels, preserved kidney function, slowed the progression to ESRD and reduced the rate of all-cause death, but it did not delay the CKD progression in long-term follow-up.	[147–150]
	Low protein diet supplemented with ketoanalogues	Reduction of substrate intake for uremic toxin generation and compensation for missing essential amino acids	A vegetarian very low protein diet supplemented with ketoanalogues alleviated uremic symptoms and deferred dialysis initiation.	[151]
	Vegetarian diet	Reduction of animal protein intake and improvement of intestinal microbiota composition and metabolism	Vegetable proteins may induce renal changes comparable to a low protein diet and prevent the proteinuric and vasodilatory effects of meat. Vegetarians or vegans had significantly lower TMAO levels than omnivores. A high-fiber diet induced the production of beneficial metabolites, such as SCFAs. IS and PCS concentrations were negatively correlated with fiber intake and positively correlated with the protein/fiber index in anuric HD patients.	[29, 152, 154, 155]
Targeting microbiota	Probiotics	Enhancement of the intestinal epithelial barrier integrity, growth inhibition of pathogenic bacteria, improvement of the host immune system and increased production of the beneficial metabolites SCFAs	Supplementation of *Faecalibacterium prausnitzii* to 5/6 nephrectomy surgery-induced CKD mice reduced plasma PCS and TMAO levels, and ameliorated renal dysfunction and inflammation. Oral administration of *Lactobacillus paracasei X11* reduced serum uric acid and renal inflammation in hyperuricemic mice. Supplementation of CKD patients undergoing HD with well-known *Bifidobacteria*, *Lactobacilli* and *Streptococc*i failed to reduce uremic toxins.	[159–161]
	Prebiotics	Growth stimulation of protective bacteria in the colon and increased production of the beneficial metabolites SCFAs	β-glucan prebiotic intervention decreased plasma IS, PCS and p-cresyl glucuronide levels. β-glucan increased *Bifidobacterium* and *Lactobacillus* and then increased the production of SCFAs.	[162–164]
	Synbiotics	Combination of probiotics and prebiotics	Synbiotic therapy reduced serum PCS but not IS and altered the intestinal microbiome in nondialysis patients with CKD stage 4 or 5.	[165]
AhR agonists and antagonists	1-aminopyrene	AhR agonist	Treatment with 1-aminopyrene activated AhR.	[91]
	Flavonoids 5',7',3',4',5'-pentahydroxy flavanone, barleriside A and rhoifolin from Semen Plantagini	AhR antagonists	Dietary 5',7',3',4',5'-pentahydroxy flavanone and barleriside A alleviated the decline in renal function and renal fibrosis in 5/6 nephrectomized rats.	[91]
	Vitamin B12 and FA	AhR antagonists	Treatment with vitamin B12 or FA rescued mice from TCDD- or FICZ-induced anemia and thrombocytopenia.	[169]
	Flavonoid baicalein from the roots of Scutellaria baicalensis Georgi	AhR agonist	Administration of baicalein significantly decreased serum uric acid and urea nitrogen to attenuate hyperuricemia and renal injury. Baicalin ameliorated aristolochic acid I-induced kidney toxicity through AhR-dependent CYP1A1/2 induction in the liver.	[170–172]
	CH223191	AhR antagonist	CH223191 normalized postischemic angiogenesis in adenine-induced CKD mice to a non-CKD level. CH223191 ameliorated CKD-induced cognitive impairment, astrocyte reactivation and neuronal activity enhancement. CH223191 did not affect cellular senescence under anoxia or reoxygenation. CH223191 prevented the increase in systolic blood pressure and diastolic blood pressure in chronic intermittent hypoxia rats.	[44, 98, 134, 136]
	Geldanamycin	Indirect AhR antagonist	Geldanamycin inhibited IS-and IAA-upregulated TF expression.	[42]
	α-NF	AhR antagonist	α-NF attenuated glomerular mesangial cell proliferation, macrophage infiltration, extracellular matrix protein deposition, cyclooxygenase /prostaglandin E2 expression, lipid peroxidation, oxidative stress, NOX activity and N-ɛ-carboxymethyl lysine formation in STZ-induced diabetic mice kidneys.	[122]
	BAY2416964	AhR antagonist	BAY2416964 alleviated cisplatin-induced kidney dysfunction and tubular injury by inhibiting cellular senescence.	[133]

*HDF* hemodiafiltration, *HD* hemodialysis, *CKD* chronic kidney disease, *ESRD* end-stage renal disease, *TMAO* trimethylamine-N-oxide, *SCFAs* short-chain fatty acids, *IS* indoxyl sulfate, *PCS* p-cresyl sulfate, *AhR* aryl hydrocarbon receptor, *FA* folic acid, *TCDD* tetrachlorodibenzo-p-dioxin, *FICZ* 6-formylindolo[3,2-b]carbazole, *IAA* indole-3-acetic acid, *CYP1A1/2* cytochrome P450 family 1 subfamily A member 1/2, *TF* tissue factor, *NOX* NADPH oxidase, *STZ* streptozotocin

### Reducing uremic toxins

Reducing circulating uremic toxins is a viable strategy for preventing or alleviating kidney diseases. Inhibiting the production and/or enhancing the clearance of uremic toxins are two rational and effective approaches [10].

#### Blood purification

Conventional HD is the main technique for reducing high concentrations of uremic toxins in the blood. HD transports solutes across a semipermeable membrane through diffusion and mainly applies to remove water-soluble small molecular-weight uremic toxins. Middle molecular-weight molecules and protein-bound uremic toxins are poorly removed [137]. Hemodiafiltration (HDF) transports solutes through diffusion and convection and effectively removes small and middle molecular-weight uremic toxins. However, HDF leads to loss of potential albumin and nutrients during treatment and the consequent need for reinfusion [137]. Adsorption-based hemoperfusion can remove middle and large molecular-weight and protein-bound uremic toxins [137]. Absorbents for hemoperfusion are usually made of polymeric resins, activated carbon, carbon nanotubes and zeolites [138]. Graphene oxide is an exceptional material because of its outstanding mechanical properties, modifiable surface functionalization and controllable interlayer distance [138]. However, the direct use of graphene oxide as an adsorbent in hemoperfusion can contribute to hemolysis and decrease blood cell and platelet levels, which may harm patients [138]. In contrast, cellulose acetate (CA) is an adsorbent material with good water and solute permeabilities and excellent hemocompatibility [138].

Improvements in the materials and production processes to increase the removal effect of uremic toxins may improve their applicability in extracorporeal purification systems. Abhishek Tyagi et al. developed a CA-functionalized graphene oxide composite material for hemoperfusion, which cleared creatinine from 83.23 to 54.87 μmol/l and uric acid from 93.4 to 54.14 μmol/l, thus restoring to normal levels within 30 min [138]. The water-dispersal adsorbent poly-β-cyclodextrin added into the dialysate can remove 96% PCS in the plasma via adsorbent once-through mode [139]. Adding poly-β-cyclodextrin cross-linked with epichlorohydrin for two hours to the dialysate can result in a twice increase in the ability to remove IS [140]. Cationic metal–organic frameworks, utilizing tetrakis ethene as a ligand skeleton, pyridyl units as functional groups, and nickel/silver nitrate as metal nodes, could almost completely remove PCS within 3 hours through anion exchange with high adsorption capacities and good adsorption kinetics [141]. In the future, optimizing sorbent materials with technical characteristics to enhance dialysis efficiency is a crucial research direction.

#### Gastrointestinal dialysis

Oral administration of cathartic compounds is a well-known method for promoting the excretion of uremic toxins and excess fluids [10]. The carbon adsorbent AST-120 has received the most attention due to its ability to absorb uremic toxin precursors in the intestinal tract and then excrete the precursors in feces, thereby reducing the absorption of uremic toxins into the blood [10]. The oral adsorbent AST-120 prevented renal accumulation of IS and PCS in adenine-induced CKD mice. However, AST-120 did not improve renal function and attenuate tubular injury and renal fibrosis in adenine-induced CKD mice [142]. Administration of AST-120 significantly decreased serum IS levels in mice with 5/6 nephrectomy-induced CKD and arteriovenous fistula. AST-120 attenuated neointima formation by inhibiting the expressions of matrix metalloproteinase (MMP)-2, MMP-9, TNF-α, and TGFβ1 in neointima tissue [143]. The therapeutic efficacy of AST-120 in CKD patients is also controversial. A multicenter, randomized, controlled trial showed that AST-120 can slow the deterioration of renal function as evidenced by inhibition of the decrease in eGFR, but it did not significantly slow disease progression in patients with moderate to severe CKD during 1 year [144]. A systematic review and meta-analysis including eight studies also demonstrated that AST-120 can effectively reduce IS levels, but controversy remained regarding slowing CKD progression and all-cause mortality [145]. So the clinical use of AST-120 for the treatment of CKD needs to be carefully considered.

#### Nutritional therapy

Nutritional therapy has been recommended for the management of patients with CKD for more than a century [146]. A diet rich in animal proteins increases populations of proteolytic bacteria that ferment dietary protein and generate uremic toxins, such as PCS, IS and TMAO. A low protein diet exhibits favorable effects on CKD progression due to the reduction of these substrates [147]. One study involving 29 healthy subjects and 20 wild-type friend leukemia virus mice revealed the influence of dietary protein intake on the mammalian metabolome. Human results showed that plasma and urinary IS levels were significantly lower, as were urinary indoxyl glucuronide, KYNA and QA, in the low protein diet group (target of 9% of total energy intake derived from protein intake) compared to the high protein diet group (target of > 25% of total energy intake derived from protein intake). The mouse results showed that the plasma p-cresyl glucuronide, phenyl sulfate and phenylacetic acid levels were decreased in the control diet (21% crude protein) compared to the high protein diet (45% crude protein). These results indicate that a low protein diet is a feasible approach for lowering uremic toxin levels in CKD patients [148]. A meta-analysis of 16 controlled trials of dietary protein restriction in CKD patients revealed that low protein intake (< 0.8 g/kg per day) or very low intake (< 0.4 g/kg per day) for 6–36 months preserved kidney function, slowed the progression to ESRD and reduced the rate of all-cause death compared to a high protein diet (> 0.8 g/kg/day) [149]. However, a large Modification of Diet in Kidney Disease (MDRD) Study revealed that a very low protein diet (0.28 g/kg/day) did not delay CKD progression and even increased the risk of death between 6 and 12 years of follow-up [150].

Due to the uncertain efficacy and potential increased risk of protein malnutrition in protein restriction regimens, the use of a low (0.6–0.8 g/kg per day) or very low (0.3–0.4 g/kg per day) protein diet is partly limited [146]. Some studies have focused on compensating for missing essential amino acids by supplementing transamination-based ketoanalogues (KA) in a low or very low protein diet. In a randomized controlled trial, 207 patients with CKD stage 4 + were allocated to a low protein diet (0.6 g/kg per day) or KA-supplemented vegetarian very low-protein diet (0.3 g/kg vegetable proteins and 0.125 g/kg KA per day). Patients on a KA-supplemented vegetarian very low-protein diet had a lower risk of reaching the composite end point (> 50% eGFR reduction or dialysis initiation) than those on a conventional low protein diet after 18 months of follow-up. A KA-supplemented vegetarian very low-protein diet also improved calcium-phosphorus metabolism and increased serum bicarbonate levels, which alleviated uremic symptoms and deferred dialysis initiation [151].

Previous dietary trials often focused on restricting total protein intake. Actually, the types of protein intake are more important, which produces a vegetarian diet [147, 152]. Vegetable proteins may induce renal changes comparable to a low protein diet and prevent the proteinuric and vasodilatory effects of meat [152]. A randomized study involving 113 healthy volunteers who were given red meat, white meat or non-meat protein (all meals prepared with 25% calories from protein) reported that chronic dietary red meat increased systemic TMAO levels by enhancing dietary precursors, increasing gut microbial TMA/TMAO production from carnitine, and reducing renal TMAO excretion [153]. The study on the oral ingestion of deuterium-labeled L-carnitine showed that vegetarians/vegans had significantly lower TMAO levels than omnivores because vegetarians/vegans had decreased gut microbiota catabolism [29]. Fiber consumption can slow CKD progression by improving the intestinal microbiota composition and reducing toxic metabolites [154]. A high-fiber diet also induced the production of beneficial metabolites, such as short-chain fatty acids (SCFAs) produced by butyrate-producing bacteria. SCFAs not only provide energy for the intestinal flora and allow the incorporation of amino acids from the colon into bacterial proteins and excretion instead of fermentation into uremic solutes, but also benefit the maintenance of intestinal epithelial functionality and integrity [154]. A prospective monocentric study using a seven-day diet record in 58 HD patients reported that IS and PCS concentrations were negatively correlated with fiber intake and positively correlated with the protein/fiber index in anuric HD patients [155].

A low protein diet, KA-supplemented diet and vegetarian diet exhibit potential benefits in reducing uremic toxin levels and slowing CKD progression. However, the benefits of these diets are often counteracted by poor patient compliance. Therefore, patient-tailored diets that reduce uremic toxins should be established for the management of CKD [10].

#### Targeting microbiota

The microbial diversity and abundance of gut bacterial species are altered in patients with CKD or AKI [156]. For example, in kidney transplant recipients, the abundances of pathogenic bacteria, including *Ruminococcacea* and *E. coli*, were increased, whereas the abundances of protective bacteria, such as *Alistipes senegalensis* and *Bacteroidales sp.*, were reduced. The metabolites of the microbiota were also significantly altered, such as a decrease in SCFAs in kidney transplant recipients [157]. Some therapies, such as dietary control as described above, and administration of probiotics, prebiotics or synbiotics, have been potential options to target the microbiome for ameliorating kidney injury and uremic toxins production [158].

A study showed that supplementation of *Faecalibacterium prausnitzii* to 5/6 nephrectomy surgery-induced CKD mice reduced plasma PCS and TMAO levels but not IS levels, and ameliorated renal dysfunction and inflammation [159]. *Lactobacillus paracasei X11* has been shown to possess excellent uric acid-lowering activity and oral administration of *Lactobacillus paracasei X11* reduced serum uric acid and renal inflammation in hyperuricemic mice [160]. However, supplementation of CKD patients undergoing HD with well-known *Bifidobacteria*, *Lactobacilli* and *Streptococc*i failed to reduce uremic toxins [161].

Prebiotics, nondigestible foods stimulating the growth of beneficial bacteria in the colon, include fructo-oligosaccharides, galactose-oligosaccharides, xylose-oligosaccharides, inulin, resistant starch, pectin, other fiber components, and milk oligosaccharides [162]. A randomized trial enrolling 59 patients with CKD stage 3–5 revealed that β-glucan prebiotic intervention decreased plasma IS, PCS, and p-cresyl glucuronide levels [163]. The study in individuals at high risk of CVD showed that β-glucan increased *Bifidobacterium* and *Lactobacillus*, increasing the production of SCFAs [164].

In practice, synbiotics are combinations of probiotics and prebiotics. Nondialysis adult participants with CKD stage 4 or 5 were recruited for a crossover trial of synbiotic therapy (combination of high molecular-weight inulin, fructo-oligosaccharides and galacto-oligosaccharides with nine different strains across the *Lactobacillus*, *Bifidobacterium*, and *Streptococcus* genera) over 6 weeks. The results showed that synbiotic therapy reduced serum PCS but not IS and altered the intestinal microbiome [165].

Regarding the mechanism of probiotics, prebiotics or synbiotics, several meta-analysis studies have shown that supplementation with probiotics, prebiotics, and synbiotics in CKD patients could decrease inflammation, improve the oxidative imbalance between pro-oxidant factors and anti-oxidant enzymes, and ameliorate the lipid profile [166]. Synbiotics enhance the integrity of the intestinal epithelial barrier and the growth of protective bacteria, inhibit the growth of pathogenic bacteria, improve the host immune system and increase the production of the beneficial metabolites SCFAs to suppress the production of uremic toxins [166, 167]. It should be noted that elevation of plasma uremic toxin levels is primarily attributed to decreased excretion due to a decline in kidney function rather than an increase in generation because culture of fecal samples from CKD patients showed no difference in PCS, indole and IAA production [168]. These results prompt a serious reconsideration of microbial manipulation as a therapeutic strategy to reduce the burden of uremic toxins.

### Developing natural AhR agonists and antagonists

Natural products are abundant and crucial sources for drug discovery. Numerous studies have revealed and considered natural AhR agonists and antagonists as alternative therapies for improving CKD and inhibiting renal fibrosis [91]. The level of 1-aminopyrene, a polycyclic aromatic hydrocarbon metabolite, was increased in the remnant kidneys of 5/6 nephrectomized rats. Treatment of RTECs with 1-aminopyrene activated the AhR, suggesting that 1-aminopyrene is an agonist of AhR [91]. Three flavonoids 5',7',3',4',5'-pentahydroxy flavanone, barleriside A and rhoifolin screened and identified from Semen Plantagini showed strong interactions with rat AhR and strong antagonistic effects on AhR activity, suggesting that they are potent AhR antagonists. Three flavonoids alleviated 1-aminopyrene-induced upregulation of profibrotic protein expression in RTECs. Dietary 5',7',3',4',5'‐pentahydroxy flavanone and barleriside A alleviated the decline in renal function and renal fibrosis in 5/6 nephrectomized rats by inhibiting AhR activation [91]. Vitamin B12 and folic acid (FA) were reported as natural antagonists of AhR. Vitamin B12 or FA deficiency in mice induced an increase in AhR transcriptional activity in the liver and accumulation of erythroid progenitors in bone marrow in an AhR-dependent manner. Treatment with vitamin B12 or FA rescued mice from TCDD- or FICZ-induced anemia and thrombocytopenia [169]. Baicalein, an important flavonoid compound isolated from the roots of Scutellaria baicalensis Georgi [170], was able to bind to AhR as predicted by molecular docking models, and induced AhR activation, indicating that baicalein is an AhR agonist [171]. Administration of baicalein (200 mg/kg) significantly decreased serum uric acid and urea nitrogen levels to attenuate hyperuricemia and renal injury [170]. The renoprotective effect of baicalein was also observed in mice with aristolochic acid nephropathy through AhR-dependent CYP1A1/2 induction in the liver [172].

Considering the double-edged sword effects of AhR in kidney diseases, the selection of AhR agonists or antagonists should be cautious and confirmed in experimental and clinical studies. However, AhR is still an intriguing and valuable therapeutic target for kidney diseases because of its important effect on renal injury and associated complications and response to uremic toxins.

## Conclusions

**Fig. 6:**
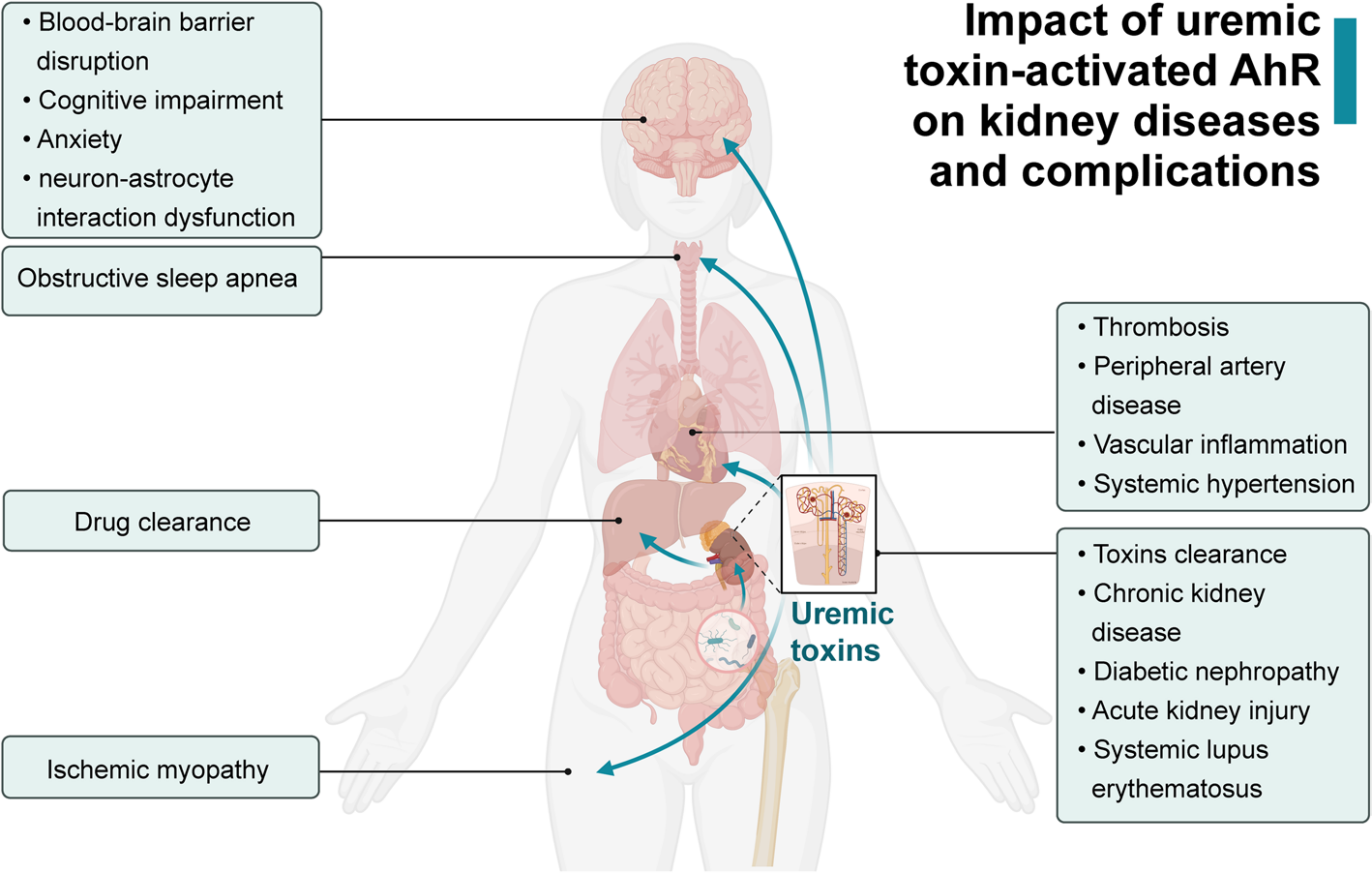
The impact of uremic toxin-activated AhR on kidney diseases and complications. Activation of AhR by uremic toxins has been implicated in various organs attributed to renal damage. In the brain, AhR activation promotes blood–brain barrier disruption, cognitive impairment, anxiety and neuron–astrocyte interaction dysfunction. AhR activation promotes the progression of systemic hypertension in obstructive sleep apnea. In drug metabolism, AhR activation facilitates drug clearance in the liver. Similarly, AhR activation in skeletal muscle exacerbates ischemic myopathy. The cardiovascular system is also impacted, with AhR activation in vessels contributing to thrombosis, peripheral artery disease, vascular inflammation and systemic hypertension. In the kidney, AhR activation promotes toxin clearance, chronic kidney disease and diabetic nephropathy and mediates acute kidney injury. AhR may be a potential marker for predicting systemic lupus erythematosus with renal damage. This figure was created with BioRendercom

As the receptor for multiple uremic toxins, AhR is elevated and activated following the accumulation of uremic toxins in the body. Accumulation of uremic toxins affects all organs and tissues, so revealing the roles of AhR activation is attracting more and more research attention. This review systematically generalizes and summarizes various functions and signaling pathways of uremic toxin-activated AhR in current nephropathy studies. Uremic toxin-activated AhR exerts detrimental biological effects on the development of CKD, CKD-associated cognitive impairment, anxiety, obstructive sleep apnea, ischemic myopathy and CVD, and DN. Uremic toxin-activated AhR increases drug and toxins clearance in CKD. In contrast, uremic toxin-activated AhR in AKI are controversial because of both protective and detrimental effects (Fig. 6). Therefore, the strategies of renal protection targeting AhR and related mechanisms, such as reducing uremic toxins or modulating AhR activation, are on the way to investigations. Uremic toxins are influenced not only by renal excretion but also by dietary intake processed in the intestinal microbiota and biotransformed in the liver, all of which can vary between individuals and may be considered targets for intervention. Although targeting uremic toxins and the AhR pathway are promising approaches, further elucidation of AhR regulation and investigations into the effects of specific agonists/antagonists are required to develop optimal therapies for human kidney disease treatment.

## Data Availability

Not applicable.

## Abbreviations

AhR: Aryl hydrocarbon receptor
CKD: Chronic kidney disease
DN: Diabetic nephropathy
AKI: Acute kidney injury
SLE: Systemic lupus erythematosus
GFR: Glomerular filtration rate
eGFR: Estimated glomerular filtration rate
PCS: P-cresyl sulfate
KYN: Kynurenine
IS: Indoxyl sulfate
Trp: Tryptophan
NFK: N-formylkynurenine
TDO: Tryptophan 2,3-dioxygenase
IDO: Indoleamine-2,3-dioxygenase
AFMID: Kynurenine formamidase
3-HK: 3-Hydroxykynurenin
KMO: Kynurenine 3-monooxygenase
KYNU: Kynureninase
3-HAA: 3-Hydroxyanthralinic acid
HAAO: 3-Hydroxyanthranilate 3,4-dioxygenase
QA: Quinolinic acid
KAT: Kynurenine amino transferase
XA: Xanthurenic acid
AA: Anthralinic acid
KYNA: Kynurenic acid
TpH: Tryptophan hydroxylase enzyme
5-HTTP: 5-Hydroxytryptophan
5-HT: 5-Hydroxytryptamine
CYP2E1: Cytochrome P450 family 2 subfamily E member 1
I3A: Indole-3-aldehyde
ILA: Indole-3-lactic acid
IAA: Indole-3-acetic acid
UV: Ultraviolet
FICZ: 6-Formylindolo[3,2-b]carbazole
I3C: Indole-3-carbinol
ICZ: Indolo[3,2-b]carbazole
IL: Interleukin
TNF: Tumor necrosis factor
FGF: Fibroblast growth factor
IAAld: Indole-3-acetaldehyde
TCDD: Tetrachlorodibenzo-p-dioxin
XAP2: X-associated protein 2
HSP90: Heat shock protein 90
ARNT: Aryl hydrocarbon receptor nuclear translocator
SRC: Steroid receptor coactivator
IKKα: IκB kinase α
DRE: Dioxin-response element
XRE: Xenobiotic-responsive element
AhRR: Aryl hydrocarbon receptor repressor
Nlrp3: Nucleotide-binding oligomerization domain, leucine rich repeat and pyrin domain containing 3
ER: Estrogen receptor
KLF6: Krüppel-like factor 6
NF-κB: Nuclear factor-κB
c-Maf: MAF bZIP transcription factor
Nrf2: Nuclear factor erythroid 2-related factor 2
DDB1: Damaged-DNA binding protein 1
Rbx1: RING-box protein 1
TBL3: Transducin-β-like protein 3
CUL4B: Cullin 4B
AR: Androgen receptor
PPARγ: Peroxisome proliferator-activated receptor γ
HIF: Hypoxia inducible factor
HDAC: Histone deacetylase
OAT: Organic anion transporter
CVD: Cardiovascular disease
ESRD: End-stage renal disease
TMAO: Trimethylamine-N-oxide
FMO3: Flavin monooxygenase 3
RTEC: Renal tubular epithelial cell
TF: Tissue factor
HUVEC: Human umbilical vein endothelial cell
MAPK: Mitogen-activated protein kinase
PAD: Peripheral artery disease
NOX: NADPH oxidase
Socs2: Suppressor of cytokine signaling 2
GLT1: Glutamate transporter 1
TGFβ1: Transforming growth factor β1
EMT: Epithelial-mesenchymal transition
EGFR: Epidermal growth factor receptor
ERK: Extracellular signal-regulated kinase
YAP: Yes-associated protein
JAK2: Janus kinase 2
PI3K: Phosphatidylinositol 3-kinase
MEK: Mitogen-activated extracellular signal-regulated kinase
miR: MicroRNA
P-gp: P-glycoprotein
T2D: Type 2 diabetes
T1D: Type 1 diabetes
STZ: Streptozotocin
COX-2: Cyclooxygenase
IR: Ischemia reperfusion
MDM2: Mouse double minute 2
EZH2: Enhancer of zeste homolog 2
OSA: Obstructive sleep apnea
CIH: Chronic intermittent hypoxia
HTN: Hypertension
HD: Hemodialysis
HDF: Hemodiafiltration
CA: Cellulose acetate
MMP: Matrix metalloproteinase
SCFAs: Short-chain fatty acids
FA: Folic acid
KA: Ketoanalogues
JNK: c-Jun N-terminal kinase
